# Association Between Donor Kidney Function and Post-Transplant Graft Function in Deceased-Donor Kidney Transplantation

**DOI:** 10.3390/jcm15030939

**Published:** 2026-01-23

**Authors:** Arefeh Sadat Pezeshk, Maximilian Nösser, Leke Wiering, Otajan Bobonov, Kim Tehyung, Brigitta Globke, Paul Viktor Ritschl, Andreas Kahl, Klemens Budde, Mira Choi, Fabian Halleck, Johann Pratschke, Robert Öllinger, Tomasz Dziodzio

**Affiliations:** 1 Department of Surgery, Berlin Institute of Health at Charité—Universitätsmedizin Berlin, Charitéplatz 1, 10117 Berlin, Germany; 2 Department of Hepatology and Gastroenterology, Berlin Institute of Health at Charité—Universitätsmedizin Berlin, Charitéplatz 1, 10117 Berlin, Germany; 3 BIH Charité Clinician Scientist Program, Berlin Institute of Health at Charité—Universitätsmedizin Berlin, Charitéplatz 1, 10117 Berlin, Germany; 4 Department of Nephrology and Medical Intensive Care, Berlin Institute of Health at Charité—Universitätsmedizin Berlin, Charitéplatz 1, 10117 Berlin, Germanymira.choi@charite.de (M.C.);

**Keywords:** kidney transplantation, glomerular filtration rate, kidney function

## Abstract

**Background/Objectives:** Donor kidney function measured by glomerular filtration rate (GFR) is widely used as a selection criterion in kidney transplantation (KT). This study addresses the knowledge gap regarding the relationship between donor GFR at organ procurement and graft function in deceased donor KT. **Methods:** We retrospectively analyzed 918 deceased donor KTs and compared donor GFRs at procurement and recipient GFRs after KT at hospital discharge and in the one-year follow-up. The Chronic Kidney Disease Epidemiology Collaboration (CKD-EPI) formula was used to estimate and compare GFRs. Donor baseline GRF was defined as the last available estimated GRF prior to organ procurement. The Kaplan–Meier analysis was used to estimate recipient and graft survival. **Results:** The median donor GFR was 92.8 mL/min/1.73 m^2^, while the median recipient GFR at hospital discharge was 37.5 mL/min/1.73 m^2^ (−60% to donor baseline, *p* < 0.001), increasing to 51.4 mL/min/1.73 m^2^ (+37%, *p* < 0.001) at one-year follow-up. One-year graft and patient survival rates were 95.3% and 98.1%, respectively. Except for grafts from donors with a GFR < 15 mL/min/1.73 m^2^ due to acute renal failure that resulted in a significantly higher delayed graft function (DGF) rate and inferior graft survival (71.4%), no correlation was observed between baseline GFRs and DGF occurrence nor graft survival. **Conclusions:** Excellent results can be achieved in KT with subnormal donor GFR. The decision to refuse a kidney offer for KT should not solely be based on donor GFR. Kidneys from donors with very low GFR (<15 mL/min/1.73 m^2^) may be transplanted, but our observation is based on a very small sample (n = 7) and should therefore be interpreted with caution, particularly given the associated higher risk of DGF and lower graft survival.

## 1. Introduction

Chronic kidney disease (CKD) is a major public health burden with an estimated global prevalence of 13.4% [1]. In patients with end-stage renal disease (ESRD) permanent kidney replacement therapy is necessary and affects up to 7 million people worldwide [1]. Kidney transplantation (KT) is the treatment of choice in these patients and has been shown to be superior to chronic hemodialysis with regard to patient survival and quality of life [2,3,4]. However, its widespread application is largely constrained by the persistent shortage of donor organs [5,6,7]. In Germany, the average waiting time for deceased donor KT is approximately 8–10 years [8,9]. Although living donor KT can partially alleviate the imbalance between organ supply and demand, the majority of KT are still performed using organs from deceased donors [10]. In the context of an aging population in Western countries and to mitigate organ scarcity, so-called marginal donor kidneys are increasingly utilized. These, predominantly include organs from older donors (≥60 years) or from donors with pre-existing conditions such as diabetes mellitus, cardiovascular disease or arterial hypertension [3,11]. Hence, these organs are more susceptible to ischemia-related injury with higher rates of delayed graft function (DGF) and primary non-function (PNF) and therefore associated with inferior transplant outcome [12].

Alongside abovementioned donor factors that affect graft outcome in the recipients, donor kidney function measured by the glomerular filtration rate (GFR) is widely accepted as the most reliable overall measure of kidney function and plays a pivotal role in donor organ selection [4,13,14]. Accordingly, reduced donor kidney function is commonly used as an exclusion criterion. While living donor transplantation allows for comprehensive pre-donation evaluation, assessment in deceased donor transplantation is often limited by time constraints and incomplete clinical information. Decisions are instead often based on the donor’s age, comorbidities and short-term kidney function. Although the impact of donor age and comorbidities has been extensively investigated on transplant outcomes, there is a scarcity of systematic evaluations focusing on GFR-based stratification of deceased donors. In this retrospective analysis, we aimed to address this knowledge gap by examining the impact of donor GFR at the time of organ procurement on recipient graft function at hospital discharge and in the one-year follow-up after deceased donor KT.

## 2. Patients and Methods

All adult (recipient age ≥ 18 years) deceased KTs performed at the Charité—Universitätsmedizin Berlin between 2010 and 2018 were analyzed. Only kidneys from deceased donors after brain death (DBD) were included and no organs from donation after circulatory death (DCD) were used. Recipients of combined organ transplants (e.g., kidney–pancreas or kidney–liver transplantation) were excluded to ensure a homogeneous study population and to avoid confounding effects related to multi-organ transplantation. Electronic records of recipient clinical data were collected from the hospital information system (SAP^®^ SE, Walldorf, Germany), the clinic’s internal database TBase 3.0 and the Eurotransplant Network Information System (ENIS). The date of the last follow-up was 1 June 2019. The study protocol was reviewed and approved by the ethics committee of the Charité—Universitätsmedizin Berlin (ID: EA4/060/17). Informed consent was waived in accordance with applicable regulations due to the retrospective character of the study. Generative AI tools (GPT-5.1) were used solely to assist with readability and to improve the language of the manuscript. All content, interpretations, and conclusions are entirely the authors’ own. Standard care immunosuppression consisted of mycophenolate mofetil, prednisolone and tacrolimus.

### 2.1. Definitions

Kidney Disease Improving Global Outcomes (KDIGO) classification was used to classify CKD and used for group comparison in the donor and recipient [15]. The Chronic Kidney Disease Epidemiology Collaboration (CKD-EPI) formula was used for GFR estimation and was calculated based on the definition of the National Health and Nutrition Examination Survey (NHANES) [14]. The Modification of Diet in Renal Disease Study Group (MDRD) GFR was additionally calculated for result illustration but not analysis purposes [16]. Donor kidney function was assessed using the last available estimated GRF prior to organ procurement and defined as baseline GRF. For analytical purposes, donor baseline GFR was categorized using conventional CKD-EPI cutoffs as pragmatic reference strata reflecting routine clinical interpretation at the time of organ allocation. Cold ischemia time (CIT) was defined as the time from cold organ perfusion during the retrieval procedure until the reperfusion of the graft in the recipient. Anastomosis time (AT) was defined as the time from the beginning of the vascular anastomosis until graft reperfusion. DGF, was defined by United Network for Organ Sharing (UNOS) criteria as the clinical manifestation of acute kidney injury, with the need for dialysis within seven days after transplantation [17,18]. PNF was defined as a permanent lack of graft function, with the need for chronic dialysis from the time of transplantation. Graft survival was calculated based on UNOS criteria, as a composite overall graft survival from the date of transplantation to the date of irreversible GF, the date of the last follow-up or to the date of patient death [19].

### 2.2. Statistical Analyses

Statistical analyses were performed using SPSS software (version 25, IBM Corporation, Armonk, NY, USA). Categorical variables are presented as frequencies and percentages and were compared using the Pearson chi-square test. Continuous variables are reported as median and interquartile range (IQR) and were analyzed using the Wilcoxon–Mann–Whitney test due to non-normal distributions. Organ survival was analyzed using the Kaplan–Meier method, with group comparisons performed using the log-rank test. Survival curves were visualized using GraphPad Prism (version 6.01; GraphPad Software, Inc.). Donor GFR values were complete for all included cases. Missing data for other variables were infrequent and attributable to the retrospective nature of the study, with data completeness exceeding 85% for the remaining variables. No formal imputation was performed, and analyses were conducted using available data only. Associations between donor GFR and recipient GFR at discharge and at one year after kidney transplantation were assessed using Spearman’s rank correlation coefficient, given the non-normal distribution of renal function parameters. All statistical tests were two-tailed, and statistical significance was defined as *p* ≤ 0.05. To identify factors associated with DGF, binary logistic regression analyses were performed. Results are reported as odds ratios (ORs) with 95% confidence intervals (CIs). Model fit was assessed using the Hosmer–Lemeshow goodness-of-fit test.

## 3. Results

**Figure 1:**
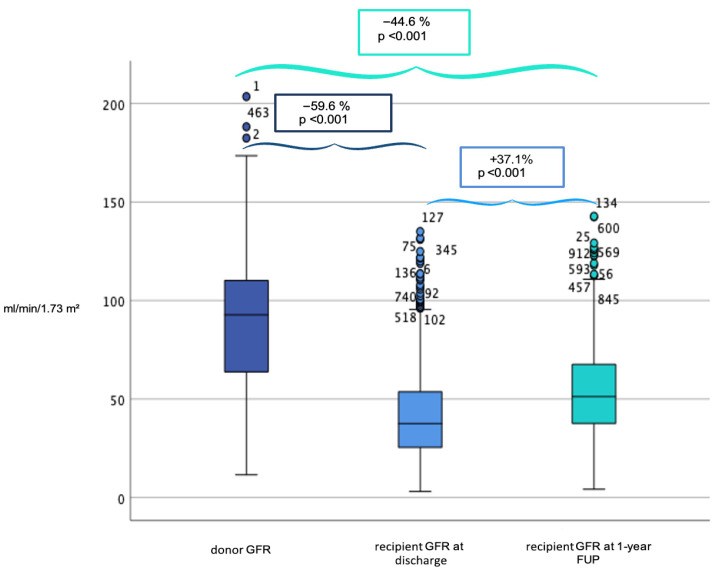
Glomerular filtration rate (GRF) values of the donor organ at organ procurement (left) compared to the GFR values in the recipient at discharge (center) and at 1-year follow-up (FUP, right), expressed in ml/min/1.73 m^2^ (CKD-EKI). Circles represent outliers (1.5–3 IQR).

A total of 918 KTs were analyzed. Of these, 478 donors were males (52.1%) with a median age of 54 years (IQR: 22 years). Organs from elderly donors (aged 60 years or older) accounted for 33% (n = 307). DGF was observed in 456 recipients (49.7%) and PNF occurred in 3 cases (0.3%). The median baseline donor serum creatinine level at organ procurement was 72.0 μmol/L (IQR: 40.6 μmol/L) with a median donor GFR of 92.8 mL/min/1.73 m^2^ (IQR: 45.5 mL/min/1.73 m^2^). Post-transplant, the median recipient GFR at discharge was 37.5 mL/min/1.73 m^2^ (IQR: 28.6 mL/min/1.73 m^2^) and improved to 51.4 mL/min/1.73 m^2^ (IQR: 30.5 mL/min/1.73 m^2^, *p* < 0.001) at the one-year follow-up (Figure 1). Donor GFR was positively correlated with recipient eGFR at discharge (Spearman’s ρ = 0.197, *p* < 0.001) and with recipient eGFR at one-year (ρ = 0.158, *p* < 0.001). Recipient eGFR at discharge was also positively correlated with 1-year recipient eGFR (ρ = 0.207, *p* < 0.001). The death-censored one-year graft survival was 95.3%, and patient survival was 98.1% (Supplementary Materials Figure S1). Detailed donor and recipient characteristics are presented in Table 1. 

### 3.1. Subgroup Analysis According to GFR

**Figure 2:**
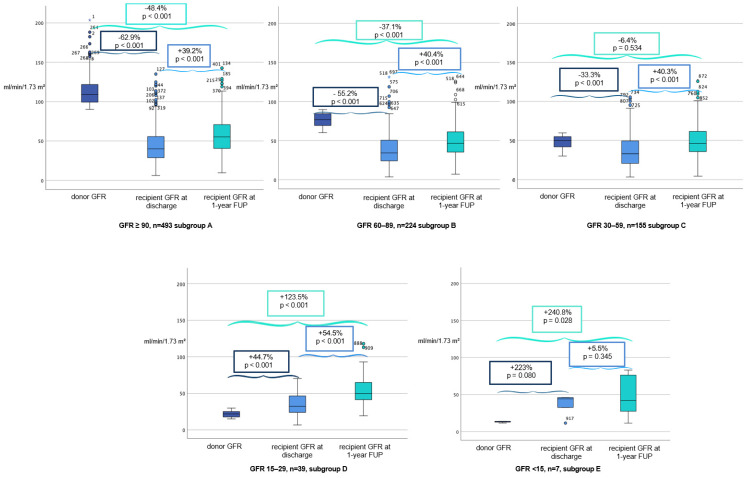
Comparison of glomerular filtration rate (GFR) course for subgroups A-E at organ procurement (left), in the recipient at discharge (center) and at 1-year follow-up (FUP, right), expressed in ml/min/1.73 m^2^ (CKD-EKI). Circles represent outliers (1.5–3 IQR), and asterisks represent extreme outliers (>3 IQR).

To compare different donor kidney functions to recipient graft outcomes, the study population was divided into five groups based on baseline donor GFR. Detailed donor and recipient characteristics for subgroups A-E are provided in Table 2 and GFR donor and recipient profiles are shown in Figure 2.

**Figure 3:**
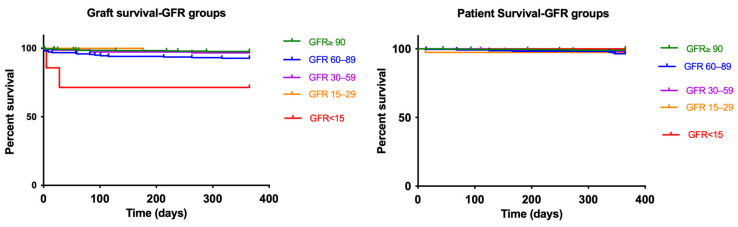
Kaplan–Meier estimates of one-year patient and graft survival by glomerular filtration rate (GFR) group.

**Subgroup A** (GFR ≥ 90 mL/min/1.73 m^2^) represented the largest cohort (53.7%) and demonstrated the best overall graft function, with a significant increase in median recipient GFR from 40.2 to 56 mL/min/1.73 m^2^ within a year (*p* < 0.001) and DGF incidence of 46.6% (Figure 2). One-year patient survival was high at 99.0%, with a 97.7% death censored one-year graft survival rate (Figure 3).

In contrast, **Subgroup B** (GFR 60–89 mL/min/1.73 m^2^), comprising 24.4% of cases, had slightly lower outcomes, with a significant increase in median recipient GFR from 34.4 to 48.3 mL/min/1.73 m^2^ within a year (*p* < 0.001) and a DGF incidence of 52.7%, highlighting a decline in function with moderate donor impairment (Figure 2). One-year patient survival was high at 96.4%, with a 92.8% death censored one-year graft survival rate (Figure 3).

In **Subgroup C** (GFR 30–59 mL/min/1.73 m^2^), donor GFR declined significantly further from 33 to 46.3 mL/min/1.73 m^2^ within a year (*p* < 0.001), but DGF incidence increased to 53.5% (Figure 2). Patient survival remained relatively stable (98.1%), and death censored one-year graft survival slightly decreased to 96.6% (Figure 3).

**Subgroup D** (GFR 15–39 mL/min/1.73 m^2^) comprised a smaller percentage of cases (4.2%) but exhibited similar trends, with a significant increase in median recipient GFR from 32.4 to 50 mL/min/1.73 m^2^ within a year (*p* < 0.001) and a DGF rate of 53.8% (Figure 2). One-year graft survival rate and death censored one-year graft survival rate were by 97.4% (Figure 3).

The poorest outcomes were observed in **Subgroup E** (GFR ≤ 14.9 mL/min/1.73 m^2^), where donor kidneys originated from acute renal failure cases (n = 7) and a non-significant increase in median recipient GFR from 44.4 to 46.8 mL/min/1.73 m^2^ within a year (*p* = 0.345) was observed. Although one-year patient survival was 100%, the graft survival rate was significantly lower at 71.4% (Figure 3). Furthermore, DGF incidence in this group was the highest at 57.1%, reinforcing concerns about delayed post-transplant recovery.

### 3.2. Subgroups Analysis According to DGF

The effects of DGF on recipient graft function were analyzed. Detailed donor and recipient characteristics by DGF are shown in Table 3.

**Figure 4:**
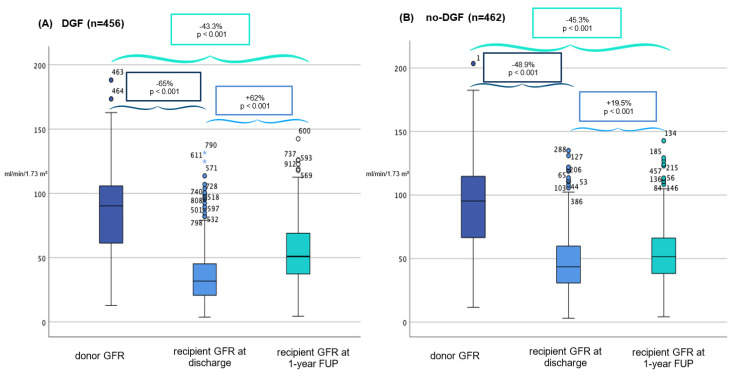
Comparison of glomerular filtration rate (GFR) course in delayed graft function (DGF; **A**) vs. no-DGF (**B**) organs at organ procurement (left), in the recipient at discharge (center) and at 1-year follow-up (FUP, right), expressed in ml/min/1.73 m^2^ (CKD-EKI). Circles represent outliers (1.5–3 IQR), and asterisks represent extreme outliers (>3 IQR).

Overall, DGF was observed in 49.7% of cases. Hypertension was the only donor characteristic that was significantly more prevalent in the DGF group (DGF: 194 [42.5%] vs. no-DGF: 151 [32.7%]; *p* = 0.006). While donor GFR did not significantly differ between DGF and no-DGF groups (DGF: 90.3 mL/min/1.73 m^2^ [IQR: 44.5 mL/min/1.73 m^2^] vs. no-DGF: 95.3 mL/min/1.73 m^2^ [IQR: 47.5 mL/min/1.73 m^2^]; *p* = 0.428), DGF recipients exhibited significantly lower GFR at hospital discharge (DGF: 31.6 mL/min/1.73 m^2^ [IQR: 24.3 mL/min/1.73 m^2^] vs. no-DGF: 43.6 mL/min/1.73 m^2^ [IQR: 30 mL/min/1.73 m^2^]; *p* < 0.001) and at the one-year follow-up (DGF: 51.2 mL/min/1.73 m^2^ [IQR: 32.7 mL/min/1.73 m^2^] vs. no-DGF: 52.1 mL/min/1.73 m^2^ [IQR: 27.7 mL/min/1.73 m^2^]; *p* < 0.001) (Figure 4). Notably, one-year patient survival remained comparable between the two groups (DGF: 98.2% vs. no-DGF: 98.5%, *p* = 0.7742), but death-censored graft survival was significantly lower in DGF patients (93.8% vs. 97.1%, *p* = 0.0167), suggesting a long-term impact of DGF on graft viability (Supplementary Materials Figure S2). In the multivariable logistic regression analysis, donor CKD-EPI categories were not significantly associated with the occurrence of DGF (*p* = 0.304). None of the other examined donor or procedural variables showed a statistically significant independent association with DGF (Table 4). Overall model fit was adequate (Hosmer–Lemeshow *p* = 0.444).

## 4. Discussion

### 4.1. Graft Function

Graft function is a key determinant of kidney transplant outcomes and is routinely assessed using estimated GFR. As low post-transplant GFR has been linked to adverse outcomes, donor GFR is commonly used as a surrogate to estimate potential graft function prior to transplantation [20,21]. While this association is well established in living donor KT, it has not yet been systematically investigated in deceased donor KT [22]. In this study we examined the association between donor kidney function and short-term graft function after KT (at hospital discharge and one-year post-KT). Further we investigated how different donor GFR levels influence the occurrence of DGF and PNF in the recipients.

### 4.2. Comparison of Donor GFR at Organ Procurement and Recipient GFR at Hospital Discharge

First, we observed that the recipient’s GFR at the time of hospital discharge was approximately 60% lower than the donor’s baseline GFR. To date, limited data are available on the course of kidney function between the donor and the recipient in deceased donor KT. Due to the lack of corresponding studies in the deceased donor KT setting, we focused on findings from living donor KT. Hawley et al. reported a 33% reduction in GFR when comparing donor and recipient GFR one week and six months after living donor KT [22]. Another study by Rinne et al. analyzed GFR changes from before transplantation to 12 months post-transplantation in 30 donor–recipient pairs. The authors observed a 30–46% lower GFR in recipients compared to donors at the one-year follow-up [23]. The greater reduction in GFR observed in our study may, at least in part, be explained by the generally more favorable conditions in living donation, including higher organ quality and shorter ischemia times, compared with deceased donor KT. In addition, because typically only one donor kidney is transplanted, the resulting reduction in nephron mass in the recipient likely accounts for a substantial proportion of the observed decrease in post-transplant GFR.

In the subgroup analysis, the decline in GFR between donor baseline and recipient GFR at hospital discharge was most pronounced in donors with a baseline GFR > 90 mL/min/1.73 m^2^ (−62.9%) and least pronounced in donors with a baseline GFR between 30 and 59 mL/min/1.73 m^2^ (−33.3%). Interestingly, recipients whose donors had a baseline GFR of <29 mL/min/1.73 m^2^ showed the opposite trend, with higher recipient GFR values at hospital discharge (>45%). This observation may be attributable to selection bias, as this donor group exhibited lower rates of comorbidities such as hypertension or diabetes. In these cases, low baseline donor GFR may have reflected temporary renal dysfunction in otherwise healthy donors. Specifically, donors with an initial GFR between 15 and 29 mL/min/1.73 m^2^ had a lower prevalence of recorded risk factors for impaired graft function and demonstrated a GFR difference of +223% between donor and recipient.

### 4.3. Natural GFR Course in the Recipient

One-year estimated glomerular filtration rate (eGFR) has been proposed as a useful surrogate marker for long-term graft durability and has been shown to be more closely associated with long-term graft outcomes than individual donor characteristics, such as donor age, or composite donor risk scores, including the Kidney Donor Profile Index (KDPI) [24]. In our analysis, kidney function at one year was relatively consistent across all donor GFR groups, with eGFR values ranging from 46.8 to 56 mL/min/1.73 m^2^. Across all groups, one-year eGFR was approximately 37% higher than eGFR measured at hospital discharge. This increase was most pronounced among recipients whose donors had an initial GFR between 15 and 29 mL/min/1.73 m^2^. This observation may in part be related to recipient and donor characteristics within this subgroup, including younger recipient age and shorter CIT. Consistent with previous reports, Serón et al. described gradual improvement in graft function during the first post-transplant year, which has been attributed to glomerular adaptation and increases in glomerular volume [25]. In contrast, recipients of kidneys from donors with a baseline GFR <15 mL/min/1.73 m^2^ showed limited improvement in GFR between hospital discharge and one-year follow-up (5.5%). Notably, this group exhibited a marked increase in GFR (223%) at the time of discharge compared with donor values, which may have constrained the extent of further functional recovery over the subsequent follow-up period.

### 4.4. Correlation Between Baseline Donor GFR, PNF and Graft Survival

In 2000, Norden et al. first demonstrated that a donor GFR <80 mL/min/1.73 m^2^ in living kidney donation is associated with a twofold higher risk of graft loss [26]. In contrast, in the study by Young et al., which included 2057 living kidney transplants, no significant difference was observed in the adjusted hazard ratio for graft loss in grafts from donors with a GFR <80 mL/min/1.73 m^2^ [27]. Although no universally accepted donor GFR threshold exists in living donor KT (reported ranges vary from 50 to 90 mL/min/1.73 m^2^), the KDIGO guidelines consider a repeatedly measured GFR <60 mL/min/1.73 m^2^ a contraindication for living donation [28]. However, this recommendation is primarily intended to protect the living donor.

In deceased donor KT, however, such recommendations do not exist due to the lack of supporting evidence. In our analysis, PNF was low across all GFR subgroups, and donor GFRs between 15 and 90 mL/min/1.73 m^2^ showed similar death-censored one-year graft survival rates ranging from 92.8% to 97.7%. Our data suggest that the use of selected donor kidneys with a GFR above 15 mL/min/1.73 m^2^ appears to be associated with acceptable short-term graft survival. In contrast, grafts from donors with a GFR <15 mL/min/1.73 m^2^ were associated with a distinctly lower one-year graft survival of 71.4%. Although patient survival in this subgroup remained high (100%), the reduced graft survival warrants careful interpretation, particularly given the small sample size (n = 7) and the overrepresentation of marginal donor organs [29]. In this context, previous studies have demonstrated that kidneys from deceased donors with acute kidney injury (AKI) can achieve graft survival comparable to that of non-AKI kidneys, suggesting that severely reduced donor renal function may in some cases reflect potentially reversible injury rather than irreversible organ damage [19]. Nevertheless, our findings indicate that kidneys with very low donor GFR values require cautious evaluation prior to transplantation, especially in older donors or in the presence of additional risk factors.

### 4.5. Correlation Between DGF with GFR, Graft and Patient One-Year Survival

An additional analysis was performed to evaluate the occurrence of DGF. Donor GFR was found to be comparable between recipients with and without DGF (DGF: 85 vs. no-DGF: 91 mL/min/1.73 m^2^, *p* = 0.474). At hospital discharge, a significant difference in GFR between the DGF and no-DGF groups was observed (31.6 vs. 43.6 mL/min/1.73 m^2^, ***p* < 0.001**). However, at the one-year follow-up, GFR values were again comparable between the groups (DGF: 51.2 vs. no-DGF: 52.1 mL/min/1.73 m^2^, *p* = 0.001). Likewise, Kim et al. reported significantly lower GFRs in recipients with DGF compared with those without DGF up to one month post-KT, but no significant differences at the three-month and one-year follow-up [30]. In the DGF group, we observed significantly lower death-censored one-year graft survival compared with the no-DGF group (93.8% vs. 97.1%, *p* = 0.0167), whereas one-year patient survival was nearly identical (98.2% vs. 98.5%, *p* = 0.7742). In 2023, Miah T. Li et al. conducted a systematic review examining the association between DGF and post-transplant outcomes, including graft failure (GF), acute rejection, patient mortality, and kidney function. They reported that DGF was associated with increased odds of graft failure (OR 3.38), acute rejection (OR 1.84), and mortality (OR 2.32) [31]. While strong evidence supports an association between DGF and impaired graft outcomes [30,32,33], the relationship between DGF and patient mortality remains debated. For example, Tapiawala et al. found that the development of DGF was associated with increased mortality at 6 and 12 months post-KT [34], whereas other studies, including a large analysis of 50,000 subjects in the US Renal Data System, found no significant effect on patient survival [30,32].

### 4.6. Limitations

Our study certainly has several limitations. First, it is a single-center, retrospective observational study that does not include data on immunological factors influencing graft function post-KT. Second, despite careful data collection, the retrospective design prevents us from fully ensuring the completeness of all data sets, which could lead to overestimated outcomes, such as higher survival or delayed graft function (DGF) rates. Potential confounding factors, including immunological risk, donor acute kidney injury and recipient comorbidities, were not systematically assessed, which is a major limitation of this study. Albeit donor and recipient GFR values were complete for all included cases, missing data for other variables were variable and attributable to the retrospective nature of data collection. Therefore, no formal imputation strategy was applied, and analyses were performed using available data only. Another important limitation is that our results are based on estimated GFR using the CKD-EPI equation, as direct assessment of donor renal function through isotopic clearance is not feasible in deceased donors. Additionally, comparisons were performed at the population level by grouping donors into predefined GFR categories rather than analyzing individual GFR trajectories. Moreover, only two short-term post-KT time points for graft function were assessed and long-term trends remain to be investigated. Due to the lack of comparable studies, we relied on data from living donations for contextual comparison. However, this approach brings its own challenges, as GFR thresholds in living donation are considerably more stringent and not directly comparable to those of deceased donors. Furthermore, transplantation conditions differ substantially between living and deceased donation, including notably shorter ischemia times in living donor KT, which further complicates direct comparisons. Finally, due to unequal group sizes and limited event numbers in some categories, multivariable modeling was not feasible, and residual confounding by donor, recipient, and procedural factors cannot be excluded. Despite these limitations, our study is among the first to explore the association between varying donor GFR levels and short-term recipient graft function in the context of deceased donor KT.

## 5. Conclusions

This study provides insights into the relationship between donor GFR and short-term graft function in deceased donor KT. Our findings highlight that recipient GFR at hospital discharge is approximately 60% lower than donor baseline GFR at procurement and improves over the first year of follow-up by approximately 37%. Grafts from donors with GFRs >15 mL/min/1.73 m^2^ demonstrated acceptable short-term graft survival rates in this cohort, whereas grafts with a GFR <15 mL/min/1.73 m^2^ showed significantly lower one-year graft survival, highlighting the importance of cautious interpretation in donor selection. The occurrence of DGF was associated with inferior graft survival, although it had no effect on patient survival at one year. Despite the limitations of this single-center, retrospective study—including reliance on estimated GFRs and a lack of immunological data—our research is one of the first studies to examine the correlation between varying donor GFR levels and short-term graft outcomes in deceased donor KT. Further research with a more comprehensive data set and direct comparison with living donations is needed to refine donor selection criteria and improve transplant success.

## Figures and Tables

**Table 1:** Donor and recipient characteristics shown as median and interquartile range (Q1–Q3) or n (%).

Variables	Total (n = 918)
Donor gender, female n (%)	440 (47.9)
Donor age (years)	54 (44–66)
Donor diabetes mellitus n (%)	66 (7.2)
Donor hypertension n (%)	345 (37.6)
Donor HCV status n (%)	14 (1.5)
Donor creatinine level (mg/dL)	0.8 (0.6–1.2)
Donor creatinine level (µmol/L)	72 (57–97)
Donor GFR (MDRD)	86.9 (60.1–121.4)
Donor GFR (CKD-EPI)	92.8 (64.4–110.0)
High-urgency transplantation n (%)	10 (1.1)
Recipient gender, female n (%)	341 (37.1)
Recipient age (years)	55 (46–65)
AT (min)	38 (30–48)
CIT (min)	637 (480–817)
Delayed graft function n (%)	456 (49.7)
Primary non-function n (%)	3 (0.3)
Recipient creatinine level dismissal (mg/dL)	1.8 (1.4–2.5)
Recipient creatinine level dismissal (µmol/L)	159 (119.6–220.1)
Recipient Creatinine 1-year follow-up (mg/dL)	1.4 (1.1–1.7)
Recipient Creatinine level at 1-year follow-up (µmol/L)	122 (100.3–153.0)
Recipient GFR at dismissal (MDRD)	35.7 (24.3–49.8)
Recipient GFR at dismissal (CKD-EPI)	37.5 (25.2–53.9)
Recipient GFR at 1-year follow-up (MDRD)	48.1 (36.1–63.0)
Recipient GFR at 1-year follow-up (CKD-EPI)	51.4 (37.9–68.3)
One-year graft survival * (%)	95.3
One-year patient survival * (%)	98.1

AT: anastomosis time; CIT: cold-ischemia time; CKD-EPI: Chronic Kidney Disease Epidemiology Collaboration formula; MDRD: Modification of Diet in Renal Disease formula; GFR: glomerular filtration rate. * estimated by Kaplan–Meier analysis.

**Table 2:** Donor and recipient characteristics for subgroups A–E comparing shown as median and interquartile range (Q1–Q3) or n (%).

Variable	Group A (GFR ≥ 90, n = 493)		Group B (GFR 60–89, n = 224)		Group C (GFR 30–59, n = 155)		Group D (GFR 15–29, n = 39)		Group E (GFR < 15, n = 7)	
Donor gender, female n (%)	297	60.2	80	35.7	48	31	12	30.8	3	42.9
Donor age (years)	51	(41–60)	62	(53–73)	59	(48–70)	46	(30–53)	61	(41–76)
Donor diabetes mellitus n (%)	24	4.9	22	9.8	19	12.3	1	2.6	0	0
Donor hypertension n (%)	158	32.0	107	47.8	69	44.5	11	28.2	0	0
Donor HCV status n (%)	8	1.6	4	1.8	2	1.3	0	0	0	0
Donor creatinine level (mg/dL)	0.6	(0.5–0.8)	1	(0.8–1.2)	1.4	(1.3–1.6)	2.8	(2.4–3.7)	3.7	(3.4–3.8)
Donor creatinine level (µmol/L)	58	(47–68)	85.1	(73.1–96.3)	123.8	(110–141.2)	248	(217–327)	319	(297–336)
Donor GFR (MDRD)	118.5	(98.1–147.8)	70.8	(64.2–77.6)	47	(38.7–52.9)	21	(16.9–24.4)	13.1	(12.3–13.9)
Donor GFR (CKD-EPI)	108.4	(99.1–121.7)	76.8	(68.9–84.6)	49.5	(41.8–54.9)	22.4	(17.8–25.5)	13.7	(11.6–14.1)
High-urgency transplantation n (%)	6	(1.2)	3	(1.3)	0	(0)	1	(2.6)	0	(0)
Recipient gender, female n (%)	140	28.4	98	43.8	76	49.0	21	53.84	6	85.7
Recipient age (years)	52	(44–61.5)	58	(49–67)	58	(49–67)	52	(44–59)	60	(53–7)
AT (min)	36.5	(29–48)	40	(30–50)	35	(29–45)	38	(32–50)	46	(45–64)
CIT (min)	643	(488–827)	660	(480–824)	603	(465–779)	544	(434–736)	611	(425–848)
Delayed graft function n (%)	230	46.6	118	52.7	83	53.5	21	53.8	4	57.1
Primary non-function n (%)	2	0.4	1	0.4	0	0	0	0	0	0
Recipient creatinine level dismissal (mg/dL)	1.7	(1.3–2.4)	1.8	(1.4–2.5)	1.9	(1.3–2.7)	2	(1.5–3.1)	1.6	(1.4–2.9)
Recipient creatinine level dismissal (µmol/L)	151.6	(117.6–209.1)	162.7	(122.0–222.8)	169.7	(118.9–238.7)	173.3	(132.6–274.0)	142.3	(120.2–258.1)
Recipient Creatinine 1-year follow-up (mg/dL)	1.4	(1.1–1.7)	1.4	(1.2–1.8)	1.4	(1.1–1.8)	1.4	(1.2–1.6)	1.3	(1.1–1.8)
Recipient Creatinine level at 1-year follow-up (µmol/L)	121.1	(99–150.3)	123.8	(103.4–158.2)	124.6	(100.3–156.9)	125.1	(105.6–143.7)	110.5	(93.7–162.6)
Recipient GFR at dismissal (MDRD)	38.2	(27.0–52.4)	33.8	(23.3–47.0)	31.1	(20.5–45.3)	30.7	(21.2–41.8)	40.6	(21.0–43.4)
Recipient GFR at dismissal (CKD-EPI)	40.2	(28.5–57.3)	34.4	(23.9–50.9)	33	(20.4–48.2)	32.4	(23.1–46.4)	44.4	(22.2–46.1)
Recipient GFR at 1-year follow-up (MDRD)	52.2	(38.5–64.9)	44.6	(34.2–58.7)	44.1	(33.2–58.4)	46.2	(37.2–60.5)	43.7	(27.2–71.2)
Recipient GFR at 1-year follow-up (CKD-EPI)	56	(41.1–71.7)	48.3	(35.4–62.8)	46.3	(35.6–61.9)	50	(40.3–65.7)	46.8	(27.5–76.4)
One-year graft survival *	97.7		92.8		96.6		97.4		71.4	
One-year patient survival *	99		96.4		98.1		97.4		100	

AT: anastomosis time; CIT: cold-ischemia time; CKD-EPI: Chronic Kidney Disease Epidemiology Collaboration formula; MDRD: Modification of Diet in Renal Disease formula; GFR: glomerular filtration rate; * estimated by Kaplan–Meier analysis.

**Table 3:** Donor and recipient characteristics comparing DGF vs. no-DGF organs shown as median and interquartile range (Q1–Q3) or n (%).

Variables	DGF (n = 456)	no-DGF (n = 462)	*p*-Value
Donor gender, female n (%)	216 (47.4)	224 (48.5)	0.735
Donor age (years)	55 (46–67)	52 (41–64)	0.253
Donor diabetes mellitus n (%)	34 (7.5)	32 (6.9)	0.756
Donor hypertension n (%)	194 (42.5)	151 (32.7)	0.006
Donor HCV status n (%)	6 (1.3)	8 (1.7)	0.475
Donor creatinine level (mg/dL)	0.8 (0.7–1.3)	0.8 (0.6–1.2)	0.191
Donor creatinine level (µmol/L)	74.3 (58–100.7)	70.7 (55.9–96.1)	0.115
Donor GFR (MDRD)	85.1 (57.9–118.3)	91 (63.1–123.2)	0.451
Donor GFR (CKD-EPI)	90.3 (61.3–105.8)	95.3 (67–114.5)	0.428
High-urgency transplantation n (%)	6 (1.3)	4 (0.9)	0.484
Recipient gender, female n (%)	160 (35.1)	181 (39.2)	0.200
Recipient age (years)	55 (46–65)	54.5 (45–65)	0.73
AT (min)	39 (30–50)	35 (28–46)	0.384
CIT (min)	663 (480–834)	607 (479–797)	0.697
Primary non-function n (%)	2 0.4	1 0.2	2
Recipient creatinine level dismissal (mg/dL)	2.1 (1.5–2.8)	1.6 (1.3–2.1)	0.172
Recipient creatinine level dismissal (µmol/L)	183.9 (135.2–251.0)	138.8 (111.8–185.6)	0.172
Recipient Creatinine 1-year follow-up (mg/dL)	1.4 (1.1–1.7)	1.4 (1.1–1.7)	0.317
Recipient Creatinine level at 1-year follow-up (µmol/L)	122 (100.8–154.7)	122 (99.9–150.7)	0.322
Recipient GFR at dismissal (MDRD)	29.8 (19.9–42.4)	41.3 (29.2–55.3)	<0.001
Recipient GFR at dismissal (CKD-EPI)	31.6 (20.5–44.9)	43.6 (30.7–60.6)	<0.001
Recipient GFR at 1-year follow-up (MDRD)	47.2 (35.7–64.7)	48.7 (36.9–60.6)	<0.001
Recipient GFR at 1-year follow-up (CKD-EPI)	51.2 (37.5–70.2)	52.1 (38.6–66.4)	<0.001
One-year graft survival *	93.8	97.1	0.0167
One-year patient survival *	98.2	98.5	0.7742

AT: anastomosis time; CIT: cold-ischemia time; CKD-EPI: Chronic Kidney Disease Epidemiology Collaboration formula; MDRD: Modification of Diet in Renal Disease formula; GFR: glomerular filtration rate. * estimated by Kaplan–Meier analysis.

**Table 4:** Multivariable logistic regression for delayed graft function (DGF; Odds ratios (OR) with 95% confidence intervals (CI) from multivariable logistic regression analysis; AKI: Acute kidney injury).

Variable	OR	95% CI	*p* Value
Donor CKD-EPI category (overall)	—	—	0.304
20–40 vs. ≥80	0.96	0.34–2.66	0.933
40–60 vs. ≥80	0.72	0.28–1.84	0.486
60–80 vs. ≥80	0.80	0.31–2.03	0.635
0–20 vs. ≥80	0.60	0.25–1.44	0.252
Donor hypertension (yes)	0.74	0.54–1.02	0.065
Donor diabetes (yes)	1.29	0.76–2.19	0.351
Donor age (per year)	1.01	1.00–1.02	0.106
Cold ischemia time	1.00	1.00–1.00	0.244
Anastomosis time	1.01	1.00–1.01	0.173
Donor sex (female)	1.04	0.79–1.38	0.777
Donor AKI	1.05	0.64–1.73	0.841

## Data Availability

The data presented in this study are available on request from the corresponding author.

## Supplementary Materials

The following supporting information can be downloaded at: https://www.mdpi.com/article/10.3390/jcm15030939/s1, Figure S1: Overall one-year patient survival estimated and overall one-year graft survival by Kaplan–Meier analysis; Figure S2: One-year patient survival for DGF and no-DGF groups and One-year graft survival for DGF and no-DGF groups estimated by Kaplan–Meier analysis.

## Abbreviations

The following abbreviations are used in this manuscript:

AKI	Acute kidney injury
AT	Anastomosis time
BMI	Body mass index
CIT	Cold ischemic time
CKD	Chronic kidney disease
CKD-EPI	Chronic kidney disease epidemiology collaboration
DGF	Delayed graft function
eGFR	Estimated glomerular filtration rate
ENIS	Eurotransplant Network Information System
ESRD	End-stage renal disease
FUP	Follow-up
GF	Graft failure
GFR	Glomerular filtration rate
HR	Hazard ratio
IQR	Interquartile range
KDPI	Kidney Donor Profile Index
KT	Kidney transplantation
MDRD	Modification of Diet in Renal Disease
NHANES	National Health and Nutrition Examination Survey
OR	Odds ratio
PNF	Primary non-function
RF	Renal function
UNOS	United Network for Organ Sharing

## References

[1] Lv J.C., Zhang L.X. (2019). Prevalence and Disease Burden of Chronic Kidney Disease. Adv. Exp. Med. Biol..

[2] Wolfe R.A., Ashby V.B., Milford E.L., Ojo A.O., Ettenger R.E., Agodoa L.Y.C., Held P.J., Port F.K. (1999). Comparison of mortality in all patients on dialysis, patients on dialysis awaiting transplantation, and recipients of a first cadaveric transplant. N. Engl. J. Med..

[3] Noble J., Jouve T., Malvezzi P., Süsal C., Rostaing L. (2019). Transplantation of Marginal Organs: Immunological Aspects and Therapeutic Perspectives in Kidney Transplantation. Front. Immunol..

[4] Laham G., Ponti J.P., Soler Pujol G. (2021). Assessing Renal Function for Kidney Donation. How Low Is Too Low?. Front. Med..

[5] Haverich A., Haller H. (2016). Organ transplantation in Germany: Critical examination in times of scarce resources. Internist.

[6] Stephan A. (2017). Organ Shortage: Can We Decrease the Demand?. Exp. Clin. Transpl..

[7] Öllinger R., Ritschl P.V., Dziodzio T., Pratschke J. (2020). Living donor kidney transplantation. Chirurg.

[8] Mayer G., Persijn G.G. (2006). Eurotransplant kidney allocation system (ETKAS): Rationale and implementation. Nephrol. Dial. Transpl..

[9] Zecher D., Tieken I., Wadewitz J., Zeman F., Rahmel A., Banas B. (2023). Regional Differences in Waiting Times for Kidney Transplantation in Germany. Dtsch. Arztebl. Int..

[10] Shepherd L., E O’carroll R., Ferguson E. (2014). An international comparison of deceased and living organ donation/transplant rates in opt-in and opt-out systems: A panel study. BMC Med..

[11] Heuer M., Zeiger A., Kaiser G., Mathé Z., Goldenberg A., Sauerland S., Paul A., Treckmann J. (2010). Use of marginal organs in kidney transplantation for marginal recipients: Too close to the margins of safety?. Eur. J. Med. Res..

[12] Chen R., Wang H., Song L., Hou J., Peng J., Dai H., Peng L. (2020). Predictors and one-year outcomes of patients with delayed graft function after deceased donor kidney transplantation. BMC Nephrol..

[13] Stevens L.A., Coresh J., Greene T., Levey A.S. (2006). Assessing kidney function--measured and estimated glomerular filtration rate. N. Engl. J. Med..

[14] Levey A.S., Stevens L.A., Schmid C.H., Zhang Y.L., Castro A.F., Feldman H.I., Kusek J.W., Eggers P., Van Lente F., Greene T. (2009). A new equation to estimate glomerular filtration rate. Ann. Intern. Med..

[15] Levin A.S., Bilous R.W., Coresh J. (2013). Chapter 1: Definition and classification of CKD. Kidney Int. Suppl. (2011).

[16] Levey A.S., Bosch J.P., Lewis J.B., Greene T., Rogers N., Roth D. (1999). A more accurate method to estimate glomerular filtration rate from serum creatinine: A new prediction equation. Modification of Diet in Renal Disease Study Group. Ann. Intern. Med..

[17] Siedlecki A., Irish W., Brennan D.C. (2011). Delayed graft function in the kidney transplant. Am. J. Transpl..

[18] Hall I.E., Reese P.P.M., Doshi M.D., Weng F.L.M., Schröppel B., Asch W.S., Ficek J.M., Thiessen-Philbrook H.M., Parikh C.R. (2017). Delayed Graft Function Phenotypes and 12-Month Kidney Transplant Outcomes. Transplantation.

[19] Liu C., Hall I.E., Mansour S., Philbrook H.R.T., Jia Y., Parikh C.R. (2020). Association of Deceased Donor Acute Kidney Injury with Recipient Graft Survival. JAMA Netw. Open.

[20] Viswanathan G., Sarnak M.J., Tighiouart H., Muntner P., Inker L.A. (2013). The association of chronic kidney disease complications by albuminuria and glomerular filtration rate: A cross-sectional analysis. Clin. Nephrol..

[21] Legendre C., Canaud G., Martinez F. (2014). Factors influencing long-term outcome after kidney transplantation. Transpl. Int..

[22] Hawley C.M., Kearsley J., Campbell S.B., Mudge D.W., Isbel N.M., Johnson D.W., May K., Preston J., Griffin A., Wall D. (2007). Estimated donor glomerular filtration rate is the most important donor characteristic predicting graft function in recipients of kidneys from live donors. Transpl. Int..

[23] Rinne A.G., Sorensen C.A., Lima S.L., Gil M.G., Mena N.N., Martín L.D., Ramírez A., Morales A., Vega N., Gallego E. (2022). Early glomerular filtration rate changes in living kidney donors and recipients: An example of renal plasticity. Clin. Kidney J..

[24] Pruett T.L., Vece G.R., Carrico R.J., Klassen D.K. (2021). US deceased kidney transplantation: Estimated GFR, donor age and KDPI association with graft survival. EClinicalMedicine.

[25] Serón D., Fulladosa X., Moreso F. (2005). Risk factors associated with the deterioration of renal function after kidney transplantation. Kidney Int. Suppl..

[26] Nordén G., Lennerling A., Nyberg G. (2000). Low absolute glomerular filtration rate in the living kidney donor: A risk factor for graft loss. Transplantation.

[27] Young A., Kim S.J., Garg A.X., Huang A., Knoll G., Treleaven D., Lok C.E., Arnold J., Boudville N., Bugeya A. (2014). Living kidney donor estimated glomerular filtration rate and recipient graft survival. Nephrol. Dial. Transpl..

[28] Chadban S.J., Ahn C., Axelrod D.A.M., Foster B.J.M., Kasiske B.L., Kher V.M., Kumar D.M., Oberbauer R., Pascual J., Pilmore H.L. (2020). KDIGO Clinical Practice Guideline on the Evaluation and Management of Candidates for Kidney Transplantation. Transplantation.

[29] Dziodzio T., Jara M., Hardt J., Weiss S., Ritschl P.V., Denecke C., Biebl M., Gerlach U., Reinke P., Pratschke J. (2019). Effects of expanded allocation programmes and organ and recipient quality metrics on transplant-related costs in kidney transplantation-an institutional analysis. Transpl. Int..

[30] Kim S.G., Hong S., Lee H., Eum S.H., Kim Y.S., Jin K., Han S., Yang C.W., Park W.Y., Chung B.H. (2021). Impact of delayed graft function on clinical outcomes in highly sensitized patients after deceased-donor kidney transplantation. Korean J. Transpl..

[31] Li M.T., Ramakrishnan A., Yu M.M., Daniel E., Sandra V., Sanichar N., King K.L., Stevens J.S., Husain S.A., Mohan S. (2023). Effects of Delayed Graft Function on Transplant Outcomes: A Meta-analysis. Transpl. Direct.

[32] Yarlagadda S.G., Coca S.G., Formica R.N., Poggio E.D., Parikh C.R. (2009). Association between delayed graft function and allograft and patient survival: A systematic review and meta-analysis. Nephrol. Dial. Transpl..

[33] Shamali A., Kassimatis T., Phillips B.L., Burton H., Kessaris N., Callaghan C. (2019). Duration of delayed graft function and outcomes after kidney transplantation from controlled donation after circulatory death donors: A retrospective study. Transpl. Int..

[34] Tapiawala S.N., Tinckam K.J., Cardella C.J., Schiff J., Cattran D.C., Cole E.H., Kim S.J. (2010). Delayed graft function and the risk for death with a functioning graft. J. Am. Soc. Nephrol..

