# Supplementary material for: Association Between Donor Kidney Function and Post-Transplant Graft Function in Deceased-Donor Kidney Transplantation
Source: J Clin Med. 2026 Jan 23;15(3):939. doi: 10.3390/jcm15030939 (PMC12898353; doi:10.3390/jcm15030939)

Suppl. Figure 1. Overall one-year patient survival estimated and overall one-year graft survival by Kaplan–Meier analysis.

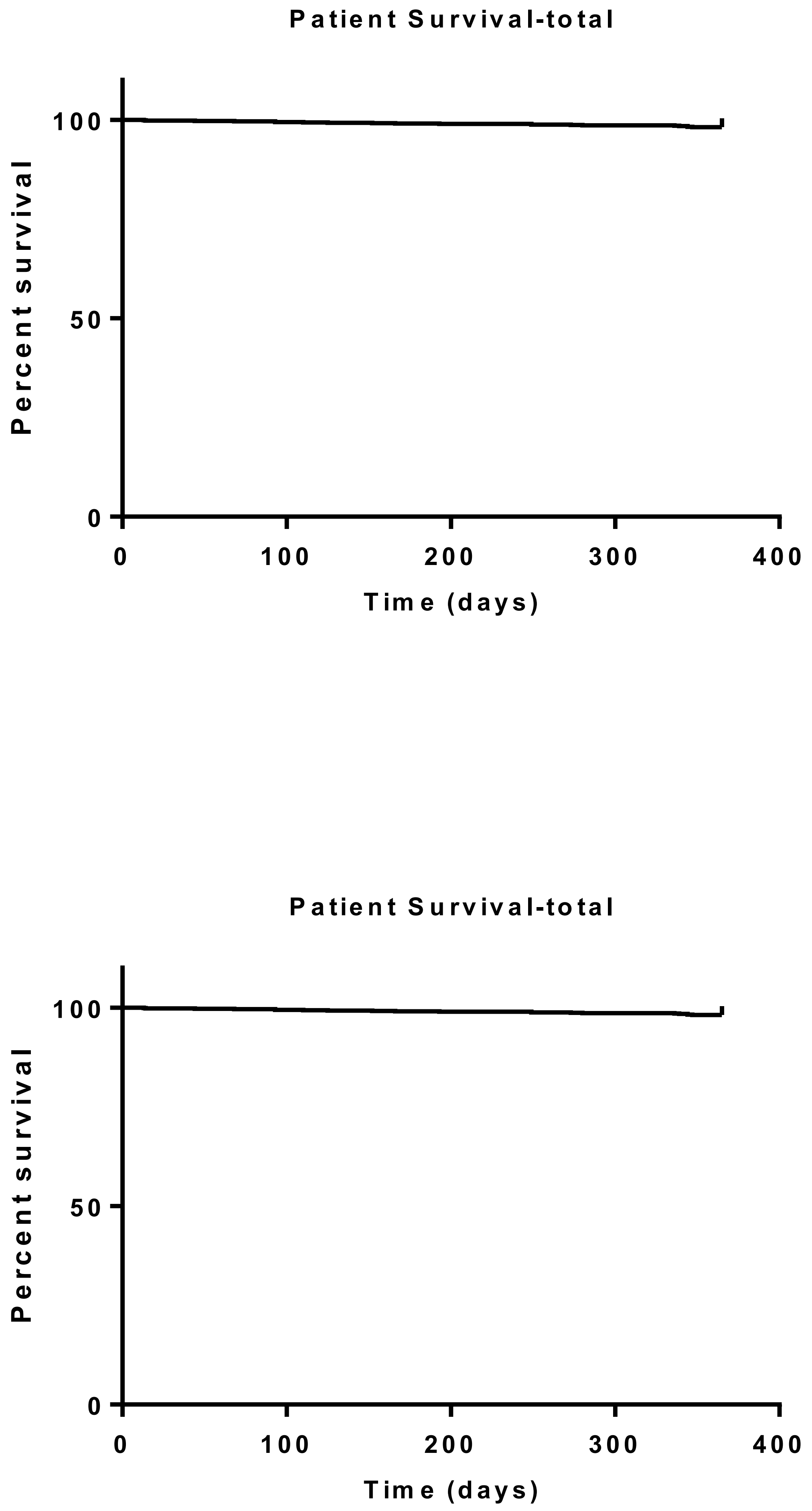

Suppl. Figure 2. One-year patient survival for DGF and no-DGF groups and one-year graft survival for DGF and no-DGF groups estimated by Kaplan–Meier analysis.

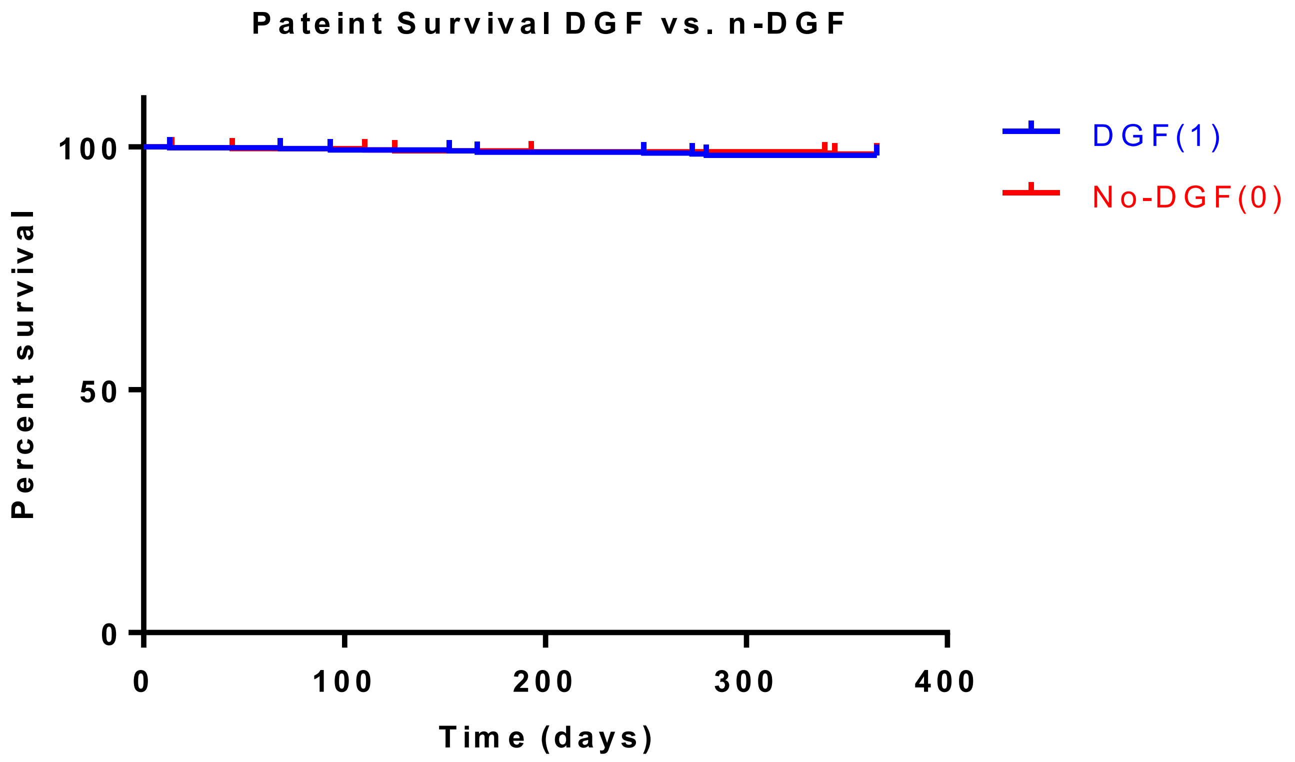

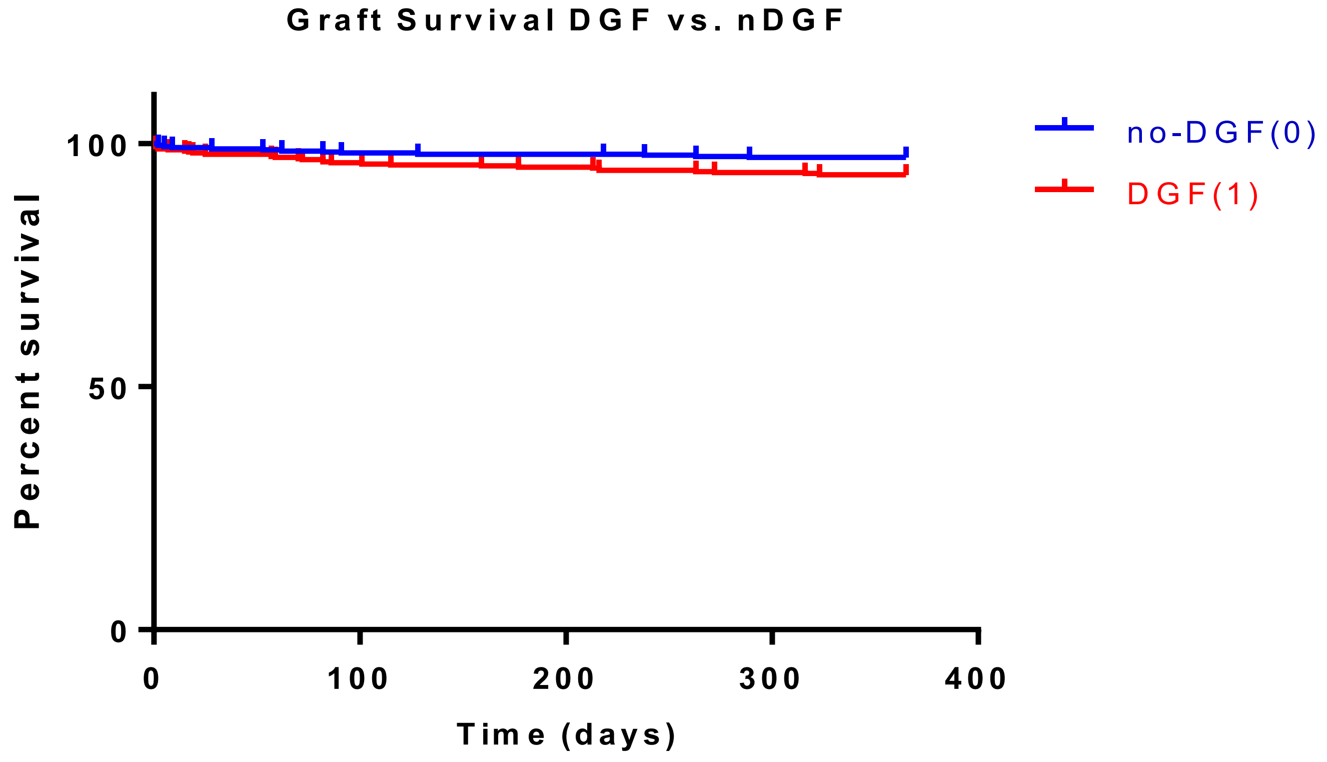

Supplement: Supplementary file 1 [file jcm-15-00939-s001.zip › jcm-4036270-supplementary.pdf]
